# Design of a Hand-Held and Battery-Operated Digital Microfluidic Device Using EWOD for Lab-on-a-Chip Applications

**DOI:** 10.3390/mi12091065

**Published:** 2021-09-01

**Authors:** Nicholas Grant, Brian Geiss, Stuart Field, August Demann, Thomas W. Chen

**Affiliations:** 1 Department of Electrical & Computer Engineering, Colorado State University, Fort Collins, CO 80523, USA; nicholasgrant314@gmail.com; 2 Department of Microbiology, Immunology, and Pathology, Colorado State University, Fort Collins, CO 80523, USA; Brian.Geiss@colostate.edu; 3 Department of Physics, Colorado State University, Fort Collins, CO 80523, USA; Stuart.Field@ColoState.EDU (S.F.); august.demann@colostate.edu (A.D.); 4 School of Biomedical Engineering, Colorado State University, Fort Collins, CO 80523, USA

**Keywords:** lab on chip (LOC), Point of Care (POC), sample preparation, digital microfluidics (DMF), Electro-wetting on Dielectric (EWOD)

## Abstract

Microfluidics offer many advantages to Point of Care (POC) devices through lower reagent use and smaller size. Additionally, POC devices offer the unique potential to conduct tests outside of the laboratory. In particular, Electro-wetting on Dielectric (EWOD) microfluidics has been shown to be an effective way to move and mix liquids enabling many PoC devices. However, much of the research surrounding these microfluidic systems are focused on a single aspect of the system capability, such as droplet control or a specific new application at the device level using the EWOD technology. Often in these experiments the supporting systems required for operation are bench top equipment such as function generators, power supplies, and personal computers. Although various aspects of how an EWOD device is capable of moving and mixing droplets have been demonstrated at various levels, a complete self-contained and portable lab-on-a-chip system based on the EWOD technology has not been well demonstrated. For instance, EWOD systems tend to use high voltage alternating current (AC) signals to actuate electrodes, but little consideration is given to circuitry size or power consumption of such components to make the entire system portable. This paper demonstrates the feasibility of integrating all supporting hardware and software to correctly operate an EWOD device in a completely self-contained and battery-powered handheld unit. We present results that demonstrate a complete sample preparation flow for deoxyribonucleic acid (DNA) extraction and isolation. The device was designed to be a field deployable, hand-held platform capable of performing many other sample preparation tasks automatically. Liquids are transported using EWOD and controlled via a programmable microprocessor. The programmable nature of the device allows it to be configured for a variety of tests for different applications. Many considerations were given towards power consumption, size, and system complexity which make it ideal for use in a mobile environment. The results presented in this paper show a promising step forward to the portable capability of microfluidic devices based on the EWOD technology.

## 1. Introduction

With the increasing computing power and shrinking size of modern microprocessors, biomedical devices have benefited tremendously from the technological advances over the years and are capable of doing more tasks in smaller packages. Researchers are able to gather data that were once limited only to the most advanced diagnostic labs. Additionally, processes that were once only available inside of central diagnostic labs are finding their way into smaller devices tailored to average users. Glucose meters may be one of the best examples of how modern technology made glucose testing readily available and easy to use for diabetic patients. Modern glucose meters can easily fit in your pocket and are battery powered for portability. The sensor technology in glucose meters is not trivial, yet they are relatively inexpensive.

Digital Microfluidics (DMF) based on an Electro-wetting on Dielectric (EWOD) is another technology offering a promising step forward in Point-of-Care (POC) devices by being able to conduct many biological assays on a micro scale. Much research has shown a range of applications for DMF such as droplet dispensing [1], electrochemistry [2], assays on human tissue [3], and cell culture [4,5]. Also included are many examples of using the EWOD technology to perform a variety of biological tasks and analyses [6,7,8].

Because DMF can perform these tasks on such a small scale, one may associate the tasks accomplished through DMF as being suitable for POC devices. A true POC device would be field deployable and not restricted to a laboratory due to the need for external benchtop equipment. Ideally, it would not require the need of an external power source and could operate using a self-contained battery pack. Some promising research has been done that suggests portability [9,10,11]; however, either the details of these systems are lacking or they were not created to be field deployable. For example, the results from [9] and [10] did not provide electrical performance of the system such as power consumption and battery life, nor did it demonstrate a real-world application using the proposed device. The device reported in [12] was perhaps one of the most complete systems with demonstrations of many real-world applications. However, it was still a large format machine, not intended for hand-held applications. Even though the authors presented a prototype photo of a hand-held device as an alternative, and no detailed information was given in [12]. 

Much of the research on DMF is focused on EWOD capabilities or demonstrating a particular application using external benchtop equipment. Little attention has been given so far to the supporting systems that drive the DMF platform. For example, EWOD requires the use of high voltage AC signals to be applied to a given electrode at a specific time to enable droplet movement. Often, the signals are created using a benchtop power supply and function generator [13]. Additionally, precise control of the droplet is through a personal computer (PC) or laptop. To improve reliability in the automated process, it is helpful for the system to understand current droplet position relative to the intended destination. Therefore, position feedback methods are implemented, however, often using an overhead camera to provide visual feedback to the control system. 

In this paper, a sample preparation device that can be used as a hand-held POC device is demonstrated. Droplet manipulation uses the EWOD technology, which is capable of moving a variety of liquid types. A removable chip is designed to store required reagents, mix appropriate volumes, and separate liquids according to the assay being performed. An integrated electronic control system consisting of on board memory, microcontroller, and high voltage drivers manages the order and timing of the droplet movement according to the assay protocol. Additionally, an impedance-based position feedback system is incorporated for reliable droplet movement. Power consumption of the entire system is kept below 100 mW. This is low enough to allow the entire portable system to be operated continuously from a lithium ion battery for up to 80 h of run time. The entire system, including the battery pack, is housed in a 109 mm × 82.5 mm × 39.7 mm package, small enough to be used as a hand-held POC device. By enabling automated sample preparation on such a small scale, the results presented in this paper demonstrate the potential of using the EWOD technology to have lab tests performed on a hand-held device and make it a step closer to reality.

## 2. Materials and Methods

### 2.1. System Architecture

**Figure 1:**
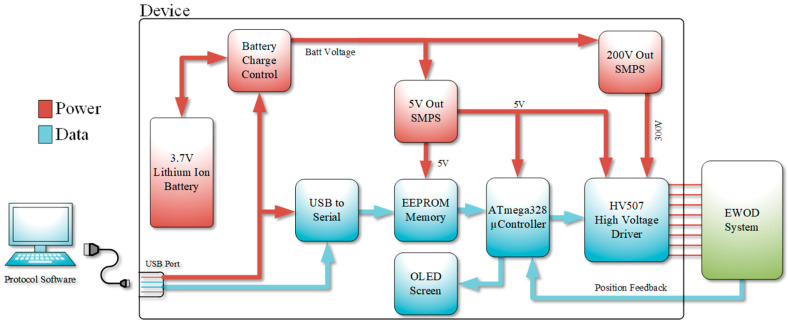
System block diagram.

To be an effective POC device, it must be portable, reliable, and versatile. The design described in this paper attempts to be effective in all areas by being battery powered, self-aware, and modular. In general, the disposable EWOD chip is loaded into the system first. The portable system contains the electronics necessary to activate the pad array according to the assay being performed. After that, the reagents and sample are loaded into a disposable EWOD chip. Figure 1 shows an overview of the device design. The main device functions and control are handled by a microcontroller (Atmel ATmega328). The device also contains 2 Mb (M95M02) Electrically Erasable Programmable Read-Only Memory (EEPROM) large enough to store a number of assay protocols such that the user can select the appropriate pre-set program for the given disposable EWOD chip. The memory allows the user to have multiple assay specific chips on hand to increase the device capability in the field. The microcontroller displays a menu of all assay protocols loaded onto the device via an Organic Light Emitting Diode (OLED) screen. Menu navigation and protocol selection are done through the on-board keypad. Once a protocol has been selected by the user, the microcontroller fetches the first sequence of the protocol from the EEPROM and transfers it to on-board high voltage drivers (HV507) which activate the appropriate pads. The microcontroller then waits for the appropriate voltage to be reached by the droplet position feedback system before fetching the next sequence from memory. Due to the manufacturing variations, the droplet feedback system adaptively controls the output voltage to the appropriate pads to ensure that the target droplet is moved to the desired location. This process is repeated until the entire protocol has been processed.

The programs stored in memory can be managed through custom software on a PC and downloaded to the device via a Universal Serial Bus (USB) port. Another microcontroller is incorporated into the system to translate the USB packets and store them into the EEPROM. Additionally, an integrated on-board power supply capable of output voltage up to 200 V is incorporated into the system to offer the capability of moving a wider range of liquid types. Finally, to maximize portability, the entire system is powered from an integrated lithium ion battery which provides up to 80 h of active run time and over 300 h in standby. The battery can be recharged via a USB port.

### 2.2. EWOD Electrode Chip for DNA Isolation and Extraction Electrode 

**Figure 2:**
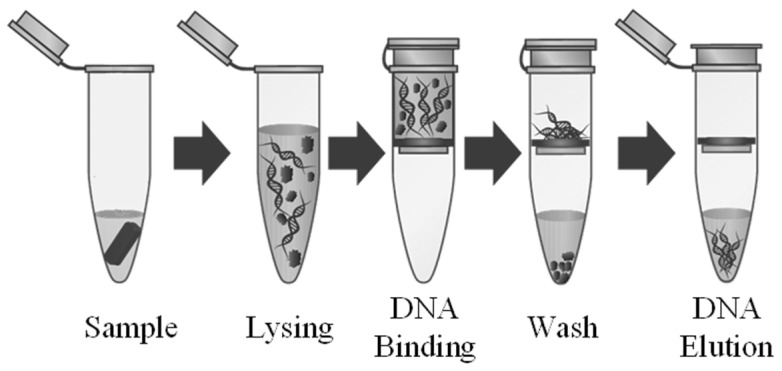
Typical DNA isolation protocol.

**Figure 3:**
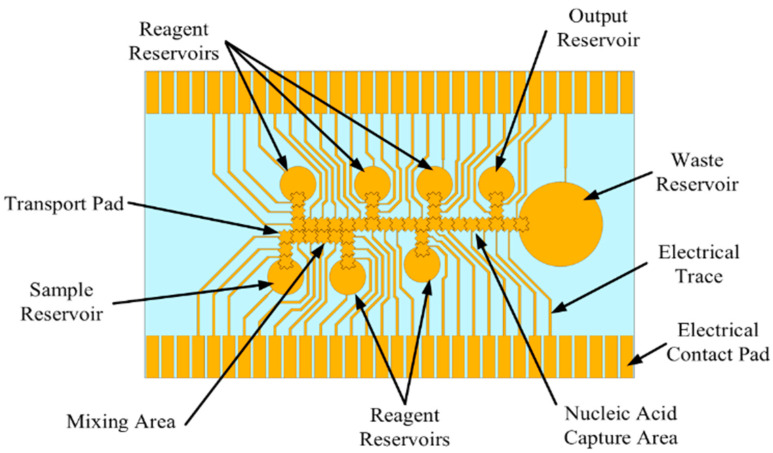
Nucleic acid extraction chip design.

For a sample preparation system to be successful in a POC device, it must be able to perform a variety of tasks so that it can support many different assays. It must also be extremely reliable such that it avoids breakdown and consistently moves liquids as desired. To evaluate the performance and practicality of such a system using the proposed self-contained and portable platform, a custom DNA isolation protocol utilizing bead binding was performed on the device. Figure 2 shows the basic DNA isolation protocol used by many commercial nucleic acid (NA) isolation kits. First, the sample is mixed with a lysing buffer to break open cells in the sample and release the DNA. Next, the DNA is forced through a filter using a centrifuge. The DNA binds to the filter but is mixed with unwanted material such as cell wall fragments, proteins, etc. A wash buffer is then run through the filter to wash away any unwanted material, leaving only the DNA bound to the filter. To release the DNA from the filter, an elution buffer is used to break the bond between the DNA and filter. The result is a solution containing only DNA from the given sample. To implement such a complex protocol, an electrode chip based on EWOD (referred to as the EWOD chip) was designed to work with the sample preparation system shown in Figure 2. Figure 3 shows the electrode pattern design for the pad array which could implement the entire NA extraction protocol.

There are 49 pads in total including the reservoir pads. There is one reservoir for a sample input, one to hold waste liquids, and one output reservoir. There are five additional reservoirs for kit reagents and a group of 10 pads intended to create a working area for liquids to be mixed. The overall process for many NA extraction kits is relatively similar to the processes described in Figure 2; therefore, this pattern will accommodate a variety of DNA extraction kits. The commercial DNA extraction kits expect a certain ratio of reagents be mixed for the kits to be effective. The ratios in the EWOD chip can be kept the same despite using a significantly smaller volume of sample and reagent. The EWOD chip conveniently makes ratio mixtures easy since the size of the transport pad dictates the volume moved. Because the size of the pads is all the same, the ratios are controlled by the quantity of droplets moved into the mixture. The transport pad size was chosen to be 1 mm by 1 mm with a saw tooth edge design. 

**Figure 4:**
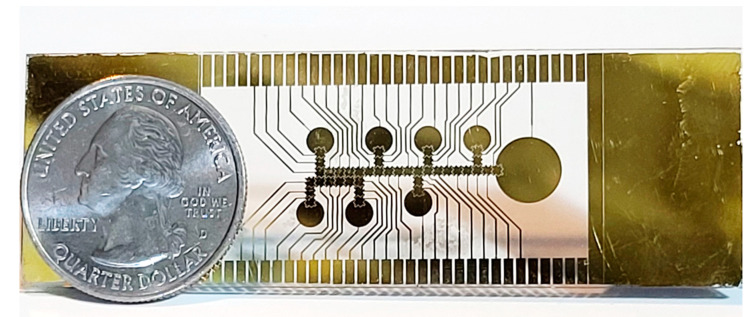
Completed EWOD chip electrodes.

An integral part of the EWOD chip design was to determine the ground plane and its distance to the electrode pads based on many factors. Ultimately, a ground plane height of 260 µm was used; however, experiments show that the device can also be operated correctly at the height of 130 µm. A higher ground plane height was found to ease the process of loading liquids. To maintain the correct height of the top plate, and to contain the desired media, two layers of 3M 468MP (130 µm thick) double sided tape was laser cut to form a perimeter around the pad array. For demonstration purposes, and to monitor system performance, the ground plane was constructed of glass with an Indium Tin Oxide (ITO) coating so that the plate remained transparent. In a production unit, the ground plane could be made of any conductive material. Holes were drilled through the glass to provide loading ports to the reservoirs. The top plate was placed on top of the double sided tape, securing it to the pad array. The EWOD electrode pattern was constructed on a 1” × 3” microscope slide using thermal evaporation. To perform the photolithography process, masks were designed in AutoCAD and manufactured by CAD/Art Services (Bend, OR, USA). A layer of Rohm Haas S1813 photoresist was applied to a glass slide using a spin coater for 5 s at 700 rpm, then 30 s at 3000 rpm. The masked photoresist was exposed to UV light for 10 s. The slides were then developed for 2 min and plasma cleaned for 2 min right before evaporation. A 20 nm layer of chromium was evaporated to the slide, followed by a 130 nm layer of gold. The lift-off process was performed by soaking the coated slide in acetone for 10 min and scrubbed clean using an acetone soaked cotton swab. Figure 4 shows the lower layer of the chip containing the EWOD pattern described above. The manufacturing steps are subject to variations creating an EWOD chip with varying electrode pad dimensions and spacing, as well as thickness of various conducting and non-conducting layers. These variations create a degree of uncertainty during the DNA isolation and extraction operation with regard to droplet movement. The system has an adaptive mechanism to determine whether a droplet has moved to a desired location and to change droplet driving voltage accordingly. This is further discussed in Section 2.3.

### 2.3. Position Feedback

**Figure 5:**
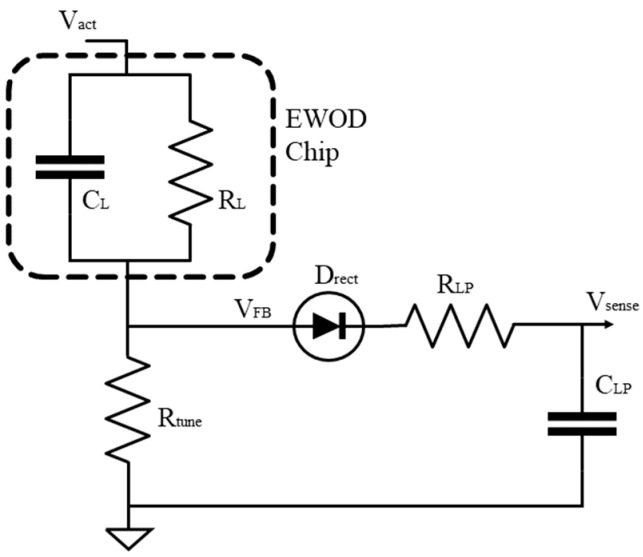
Position feedback circuit.

**Figure 6:**
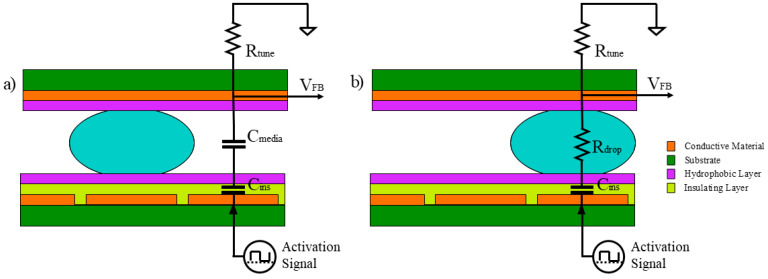
Droplet position equivalent circuit. (**a**) droplet not over the active pad; (**b**) after droplet has reached the active pad.

To make droplet movement more reliable, an impedance based feedback detection circuit (Figure 5) was implemented to monitor the intended droplet movement. The selected design is similar to those described elsewhere [13,14]. The EWOD system inherently produces a different impedance based on the presence of the droplet over the active pad. As seen in Figure 6, the EWOD chip forms a voltage division circuit between the activated pad and R_tune_. When the droplet has not reached the active pad, a high impedance path is formed through the capacitive nature of the media (C_media_). The relatively high impedance of C_media_ causes a large voltage drop across C_media_ and C_ins_ forcing V_FB_ to be low (effectively ground). As the droplet reaches the active pad, it displaces the media, removing C_media_ and inserting a low resistive path formed by the conductive droplet (R_drop_). The low resistance of the droplet causes a larger voltage drop across R_tune_. Because the capacitance of C_media_ is dependent on the geometry of the EWOD chip (pad size, ground plane height, etc.) R_tune_ is selected to produce a max voltage that is within the range of the sensing Analog to Digital Converter (ADC) of the system. The signal seen at V_FB_ is an attenuated version of the activation signal with a 0 V direct current (DC) offset due to the insulating layer of the EWOD chip (C_ins_) forming a high pass filter. Therefore, V_FB_ is sent through a half wave rectifier and low pass filter (Figure 5) to create a DC voltage (V_sense_) that is proportional to the droplet position. 

The microcontroller monitors V_sense_ to determine if the droplet has reached the intended destination, and able to progress to the next sequence in the protocol. The feedback system detects whether a droplet fails to move on to the active pad. If so, the applied voltage for the movement is adjusted and the movement is tried again until the droplet moves on to the active pad, or the maximum number of trials have been reached. Common situations for a droplet failing to be moved on to an active pad include dirt particles in the EWOD system, manufacturing variations resulting in surface imperfections, and dielectric breakdown during operation. 

### 2.4. Supporting Circuits

**Figure 7:**
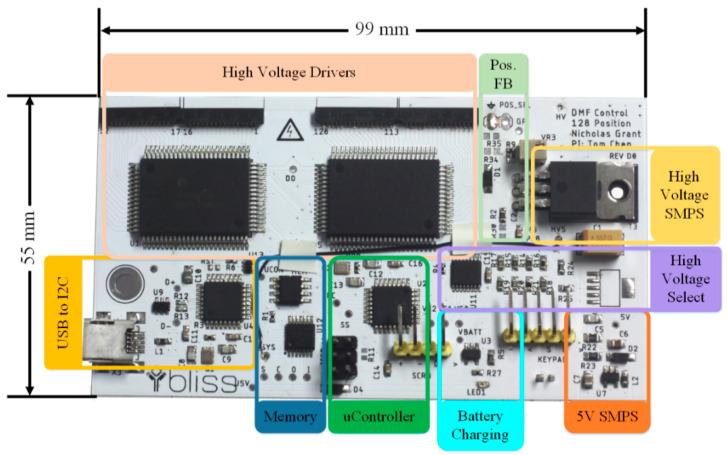
PCB layout.

**Figure 8:**
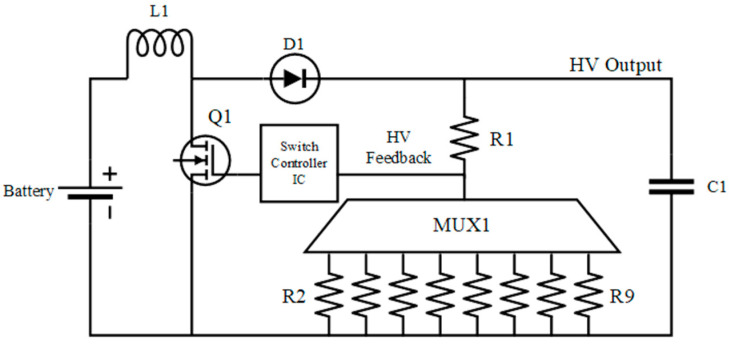
Adjustable high voltage SWPS.

The custom-designed FR-4 2-layer printed circuit board (PCB) (99 mm × 55 mm), shown in Figure 7 contains the high voltage drivers, power regulation, and control circuits. An additional custom 4-layer PCB, referred to as the interface PCB, was designed to act as an interface to the EWOD system which transfers all 128 high voltage signals to their respective pads on the chip. Battery voltage is connected to each Switched Mode Power Supply (SMPS) DC–DC boost converter. One SMPS provides 5 V to the digital control components while another SMPS supplies the high voltage required for droplet movement. The SMPS that provides the 5 V logic supply voltage is controlled by the power boost regulator (LM2735YMF), which has an integrated switching metal–oxide–semiconductor field-effect transistor (MOSFET), external diode, and inductor. The EWOD system requires a minimum of 60 V to initiate droplet movement. A custom DC–DC boost converter, shown in Figure 8, was developed to produce adjustable voltages up to 200 V from the battery voltage. The high voltage SMPS is controlled via a DC–DC controller integrated circuit (IC) (MAX1771) where the high voltage feedback signal is selectable to change the voltage magnitude. Due to the high voltage, the capacitor, inductor, and switching MOSFET (IRF644PBF) are external from the controller IC. Two high voltage serial to parallel drivers (HV507PG) generate the high voltage waveforms and direct them to the EWOD system via the adapter PCB. The interface PCB board is connected to the main controller board through eight of the DF52-16S-0.8 connectors.

### 2.5. User Control and Software Interface

User control of the device is through a four button keypad, OLED screen, and custom built user interface software on a host computer (PC). In general, the user will connect the device into a PC using a USB port. Once the software detects the communication channel, the on-board memory can be accessed. Files on the PC contain the sequence of pads to be executed to perform the required sample preparation. The user, via the custom software, selects the protocols to be conducted off-line, and downloads them to the on-board memory of the device. Once transfer of data is complete, the device is ready to be used in the field without PC. The user can recall any protocol previously downloaded into device memory via the OLED screen and the keypad. Protocols downloaded to the device are stored in the onboard 2Mbit EEPROM (M95M02-DRMN6TP) via the USB to serial microcontroller (Atmel ATmega16U2), which translates the USB data into SPI for the EEPROM. The USB to SPI microcontroller is seen as a virtual COM port to the PC where supporting libraries are standard in current Windows operating systems. The main microcontroller (ATmega328) handles user input from the keypad and data output to the OLED screen.

**Figure 9:**
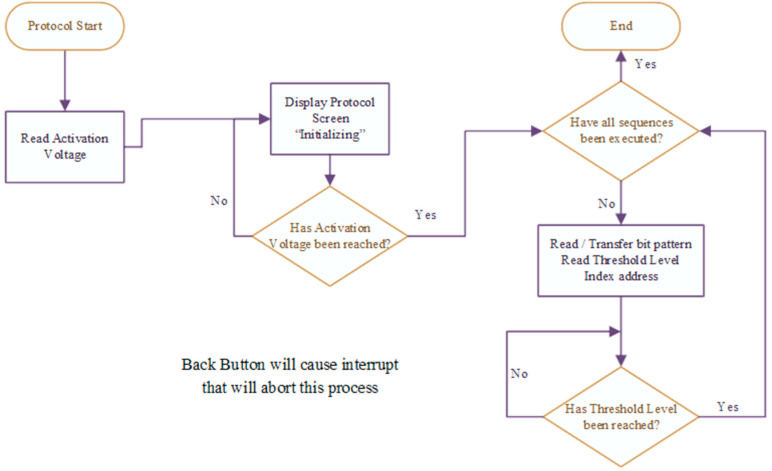
Protocol control flow.

To keep the firmware of the main microcontroller light, the PC software populates the EEPROM with a specific format such that the protocols can be called back easily by the microcontroller. When the device is first powered on, a splash screen is displayed while the microcontroller sets up its peripherals. Once ready, the Protocol Select menu is displayed where the user can navigate to the desired protocol. During execution of the selected protocol, the display shows protocol progress. Additionally, the user can stop the active protocol through the keypad, where the user is prompted to confirm the request. If the request to cancel the protocol is confirmed, the user is taken back to the Protocol Select menu. Figure 9 shows the control flow once a stored protocol has been selected.

**Figure 10:**
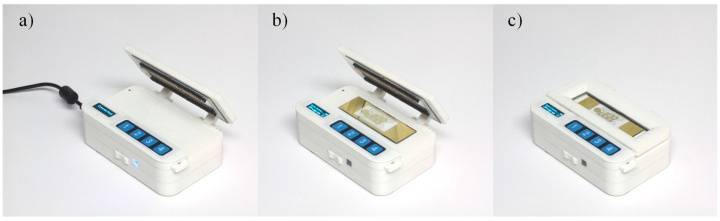
Device implementation (**a**) Charging/PC connected; (**b**) Open; (**c**) Closed.

A custom case was designed (109 mm × 82.5 mm × 39.7 mm) to contain the battery and the electronics (Figure 10). The top of the case features a clamshell style locking clamp that secures the EWOD system during operation and allows EWOD chips to be easily changed. The OLED screen and four button keypad of the user interface are integrated into the top. Located on the side of the case are the USB port and the charging indicator light.

### 2.6. EWOD System Preparation

The preparation of the EWOD system was based a custom method which uses Chitosan tagged magnetic beads to capture the target DNA. Magnetic beads have been used successfully in lab-based DNA extraction protocols [15,16]. To prepare the chip for the magnetic bead protocol, the chip is filled with 50 µL of 1cSt silicone oil. A fluorescently tagged DNA with an initial concentration of 1 µg/µL was diluted 10:1 in DI water. In addition, 2 µL of the tagged DNA was loaded into the sample reservoir. An additional reservoir was loaded with the magnetic beads which were in an MES buffer solution. To control the pH levels, and therefore binding and eluting of DNA, MES buffer (pH 5.0) was used to bind DNA to the beads. Tris buffer (pH 8.8) was used to elute the DNA from the beads. Furthermore, 2 µL of Tris buffer were loaded into a separate reservoir to act as the elution buffer.

### 2.7. DNA Isolation

**Figure 11:**
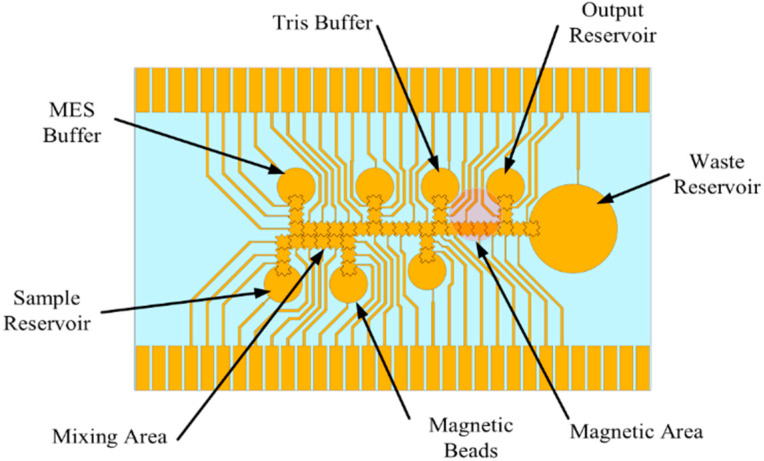
Magnetic bead EWOD chip layout.

The same EWOD chip design as shown in Figure 3 was used, but the reservoirs were simply filled with the appropriate liquids (Figure 11). A program was written to accommodate the activation sequences in the protocol, and an embedded magnet was incorporated into the device design. To help ensure that the environment became acidic, a 2:1 volume of MES buffer was mixed with the sample DNA. The MES (2-(N-morpholino) ethanesulfonic acid) buffer, sample DNA, and magnetic beads would be mixed at the appropriate ratios and then transported to the magnetic area to let the DNA bound beads precipitate from the surrounding supernatant. Once separated, the supernatant containing any unbound DNA is moved to the waste reservoir and discarded. Tris buffer is then moved through the magnetic area to elute the DNA from the beads, while leaving the beads in the magnetic area.

## 3. Results and Discussion

### 3.1. High Voltage Power Supply

**Figure 12:**
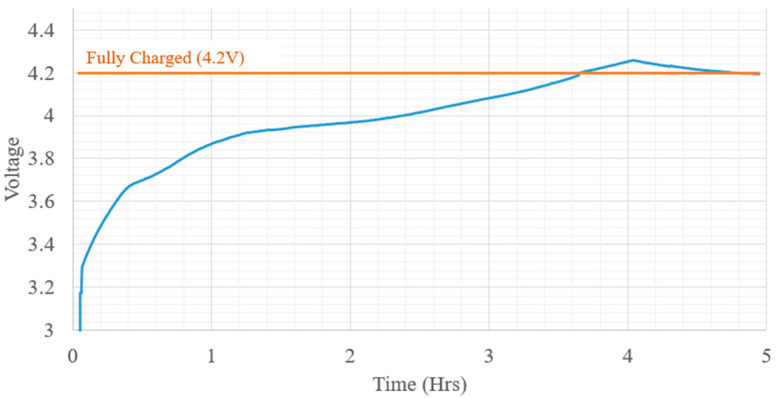
Battery charge waveform.

The battery discharge characteristics were obtained by discharging it at a constant current of 450 mA, which would closely represent the estimated max system current draw during operation. Lithium-ion batteries require a specific charging sequence, where a constant current is supplied until a specific battery voltage has been reached. A constant current of 500 mA (¼ C for the selected battery) is delivered to the battery until it reached the fully charged voltage of 4.2 V. After the fully charged voltage is reached, the charging controller changes to a constant voltage circuit to maintain the 4.2 V. As seen in Figure 12, during testing of the charge controller, slight overshoot was detected when the max charge voltage was reached. However, it remained inside of the controller’s specifications and within the built-in battery protection circuits. 

**Figure 13:**
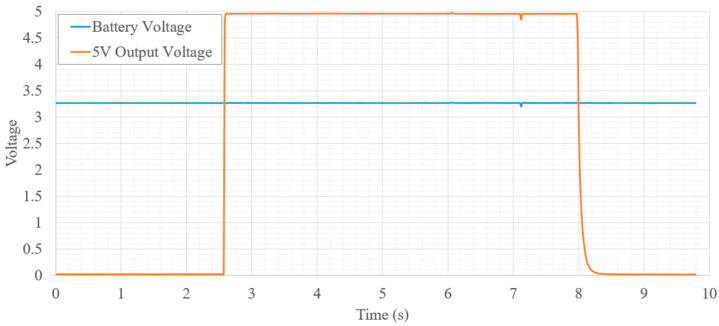
5 V SMPS power on/off waveforms.

**Figure 14:**
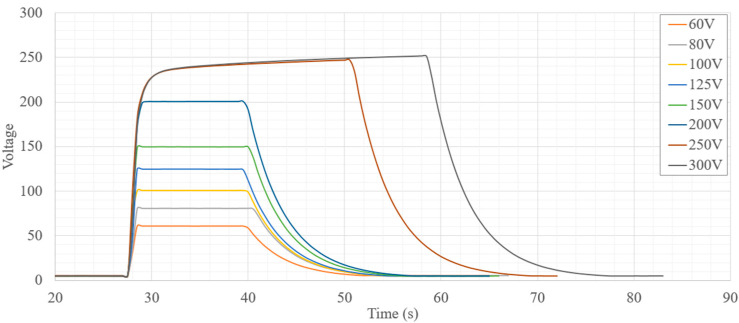
High voltage SMPS power on/off waveforms.

**Figure 15:**
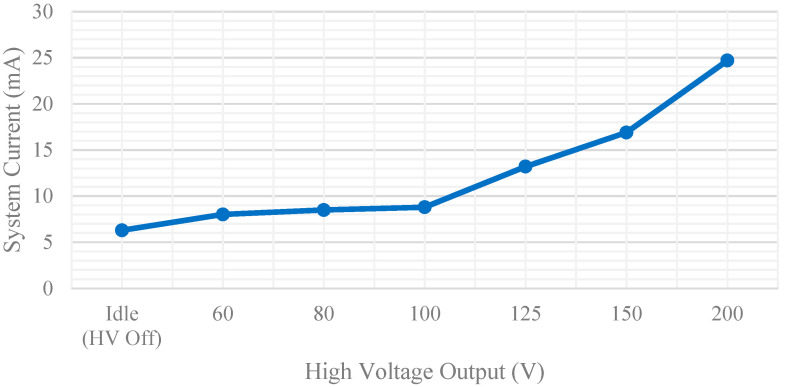
System current consumption for different activation voltage levels.

Both battery voltage and boost converter output voltages were monitored as the system was powered on and off. Figure 13 shows the output voltage of the 5 V switch mode power supply (SMPS) as the system is powered on and off. It can be observed that the boost converter quickly reaches the correct output voltage with no overshoot or ringing. Additionally, when the device is switched off, the voltage quickly drops to zero with minor RC settling time near 0.5 V. The high voltage boost converter was also recorded during enabling and disabling of the high voltage circuits. Along with capturing the rise and settling time of each voltage level, the maximum voltage capability was also observed as seen in Figure 14. All voltage levels up to 200 V were reached within 2 s. System power is also dominated by the HV circuits and the system current was measured for each of the high voltage settings. Figure 15 shows the total system current draw for the different high voltage output settings. It is noted that the efficiency of the SMPS diminishes quickly after 100 V and starts decreasing exponentially as output voltage increases. 

### 3.2. Adaptive Activation Control from Droplet Position Feedback

**Figure 16:**
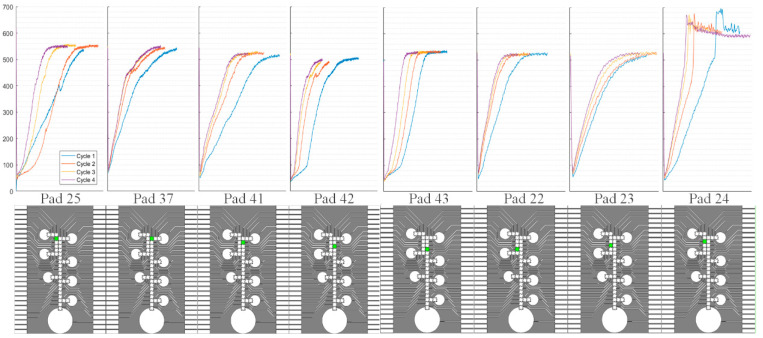
Position feedback V_sense_ plots.

The capability of the feedback circuit was demonstrated by recording the values of V_sense_ while moving a droplet in a loop over a select group of pads. Figure 16 shows the recorded values of V_sense_ over time by the analog-to-digital converter of the microcontroller as droplet was cycled around the loop manually four times. The location of each pad shown in Figure 16 is color coded green in the corresponding diagram below each captured V_sense_ waveform. Most of the pads show ideal results where the magnitude of V_sense_ asymptotes to the desired value as the droplet moves over the active pad. Additionally, on each pass of the loop, the same threshold voltage is reached. Pad 24 highlights possible damage to the pad resulting in a slightly higher voltage seen on V_sense_. Since most of the voltage drop from the active pad is over the droplet and dielectric layer, any damage caused by breakdown lowers the resistance, which raises the voltage over Rtune. Some pads, such as 41, show a slower rise to its max voltage during the early cycles, and droplet movement becomes faster with every additional cycle. This implies that the droplet is becoming easier to move with each use. Longer movement time is a result of higher resistive forces on that pad possibly due to surface imperfections. Pads 25 and 37 have slightly higher max voltages which could also be caused by manufacturing variations. Overall, these results provide insight into many variations the system must face. This information was then used to create an adaptive algorithm in firmware to help reliably move droplets.

### 3.3. DNA Isolation

**Figure 17:**
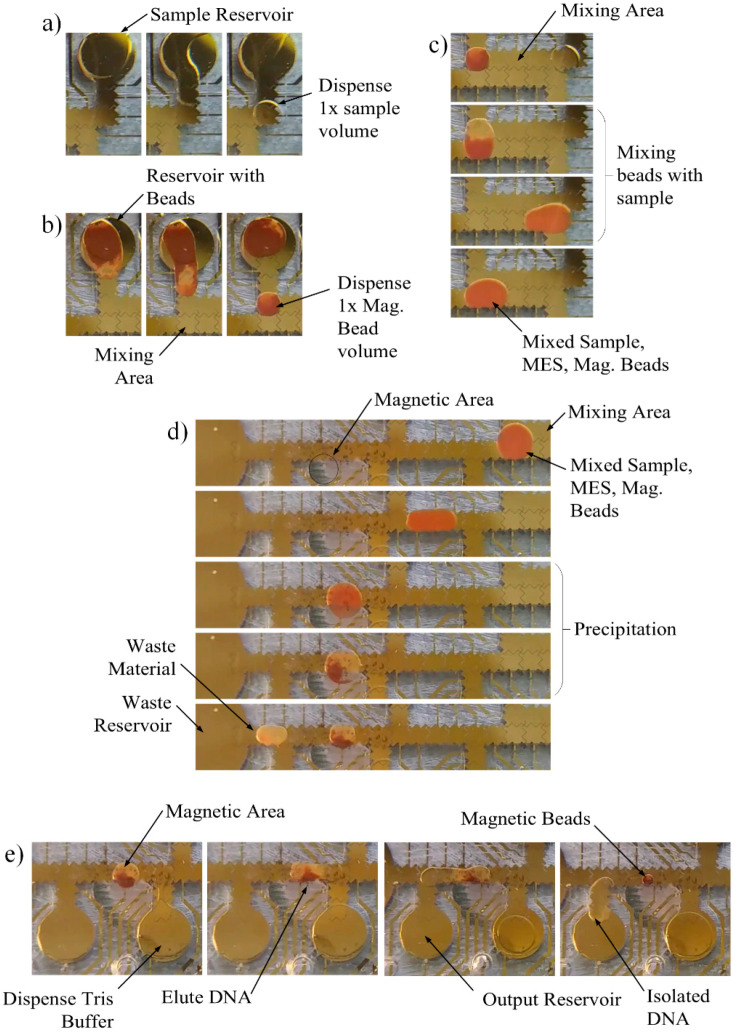
Bead method DNA isolation sequences. (**a**) Move sample to mix area; (**b**) Move beads buffer to mix area; (**c**) Mix sample and beads; (**d**) Move mixture to capture area; (**e**) Elute beads and move to output.

DNA was isolated using a custom magnetic bead-based protocol described in the Materials and Methods section above. Using the same EWOD system described, buffers and sample were loaded into the reservoirs using a pipette. A program containing the appropriate sequences to complete the protocol was loaded onto the device and executed. One volume of sample was moved to the mixing area (Figure 17a). Next, the magnetic beads were moved to the mixing area and mixed with the sample (Figure 17b,c). Finally, the full volume of the mixture was moved over the magnet in the capture area (Figure 17d). After letting the beads settle over the magnet, excess liquid was moved to the waste reservoir, leaving bound DNA over the magnet. Finally, the elution buffer was moved through the capture area into output reservoir (Figure 17e).

**Figure 18:**
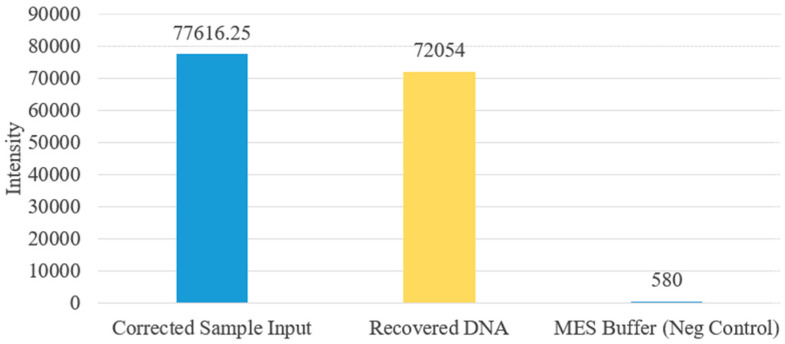
Plate reader plot.

**Figure 19:**
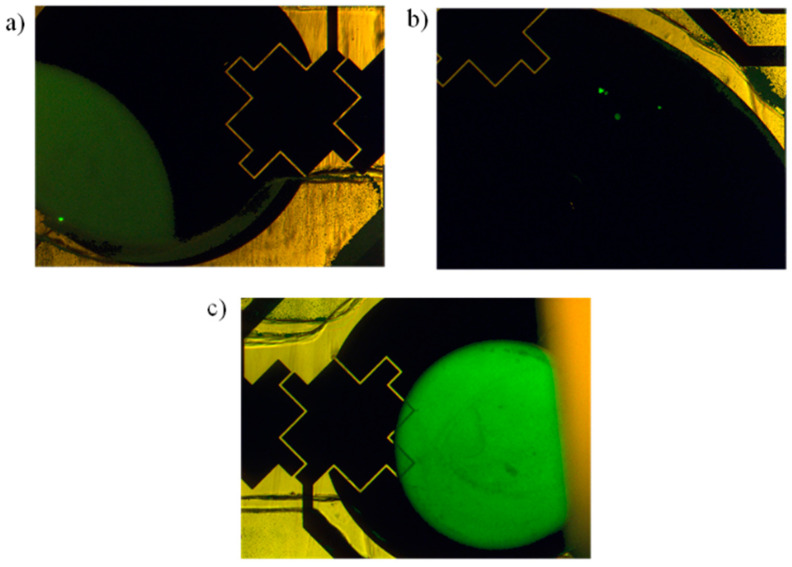
UV microscope images. (**a**) sample DNA; (**b**) waste reservoir; (**c**) recovered DNA in output reservoir.

The output liquid was removed using a pipette and placed into a plate reader. The output results were compared against 1 µL of the original sample DNA and MES buffer. Since the protocol used only 260 nL of DNA sample, the results of the sample DNA were divided accordingly to correct for the difference in volume. Figure 18 shows the plate reader results for the magnetic bead protocol. The intensity of the sample DNA was 77,616 while the intensity of the output was 72,054, suggesting a recovery of 92.8%. The high recovery rate correlates well with the fluorescent images shown in Figure 19. Negligible fluorescents were seen in the waste reservoir, and no beads were observed in the output reservoir.

## 4. Conclusions

This paper presents a hand-held, fully integrated, EWOD system platform that can be used for POC applications. The initial results illustrate that our fully self-contained and portable system is capable of moving a variety of liquids to complete a DNA isolation protocol on chip. An electronic control system consisting of on-board memory, microcontroller, and high voltage drivers adaptively manage the order and timing of the droplet movement according to the assay protocol. Power consumption is kept low enough to allow sample preparation operations using a lithium ion battery. The entire system size is kept small enough to be easily integrated into a hand-held POC device. By enabling automated sample preparation on such a small form factor, we have demonstrated the potential of having lab tests performed on a field deployable device in the future. Our future efforts include expanding the use of the system to other sample preparation applications while collecting sufficient data in each application to examine the practical use of the system in terms of its variability and a potential model/method for statistical data analysis in practice.

## References

[1] Ren H., Fair R.B., Pollack M.G. (2004). Automated on-chip droplet dispensing with volume control by electro-wetting actuation and capacitance metering. Sens. Actuators B Chem..

[2] Karuwan C., Sukthang K., Wisitsoraat A., Phokharatkul D., Patthanasettakul V., Wechsatol W., Tuantranont A. (2011). Electrochemical detection on electrowetting-on-dielectric digital microfluidic chip. Talanta.

[3] Mousa N.A., Jebrail M.J., Yang H., Abdelgawad M., Metalnikov P., Chen J., Wheeler A.R., Casper R.F. (2009). Droplet-Scale Estrogen Assays in Breast Tissue, Blood, and Serum Movie S1 (wmv format). Sci. Transl. Med..

[4] Aijian A.P., Garrell R.L. (2015). Digital Microfluidics for Automated Hanging Drop Cell Spheroid Culture. J. Lab. Autom..

[5] Zhai J., Li H., Wong A.H.H., Dong C., Yi S., Jia Y., Mak P.I., Deng C.X., Martins R.P. (2020). A digital microfluidic system with 3D microstructures for single-cell culture. Microsyst. Nanoeng..

[6] Vergauwe N., Witters D., Ceyssens F., Vermeir S., Verbruggen B., Puers R., Lammertyn J. (2011). A versatile electrowetting-based digital microfluidic platform for quantitative homogeneous and heterogeneous bio-assays. J. Micromech. Microeng..

[7] Srinivasan V., Pamula V.K., Fair R.B. (2004). An integrated digital microfluidic lab-on-a-chip for clinical diagnostics on human physiological fluids. Lab Chip.

[8] Shen H.H., Fan S.K., Kim C.J., Yao D.J. (2014). EWOD microfluidic systems for biomedical applications. Microfluid. Nanofluidics.

[9] Gong J., Fan S.-K., Kim C.J. Portable digital microfluidics platform with active but disposable Lab-On-Chip. Proceedings of the 17th IEEE Interitional Conference on Micro Electro Mechanical Systems.

[10] Joshi K., Velasco V., Esfandyarpour R. (2020). A low-cost, disposable and portable inkjet-printed biochip for the developing world. Sensors.

[11] Jang L.S., Hsu C.Y., Chen C.H. (2009). Effect of electrode geometry on performance of EWOD device driven by battery-based system. Biomed. Microdevices.

[12] Sista R., Hua Z., Thwar P., Sudarsan A., Srinivasan V., Eckhardt A., Pollack M., Pamula V. (2008). Development of a digital microfluidic platform for point of care testing. Lab Chip.

[13] Fobel R., Fobel C., Wheeler A.R. (2013). DropBot: An open-source digital microfluidic control system with precise control of electrostatic driving force and instantaneous drop velocity measurement. Appl. Phys. Lett..

[14] Shih S.C.C., Fobel R., Kumar P., Wheeler A.R. (2011). A feedback control system for high-fidelity digital microfluidics. Lab Chip.

[15] Abdulwahab S., Ng A.H.C., Chamberlain M.D., Casper R.F., Wheeler A.R. (2017). Lab on a Chip Towards a personalized approach to aromatase inhibitor therapy: A digital microfluidic platform for rapid analysis of estradiol in core-needle- biopsies. Lab Chip.

[16] Hung P.-Y., Jiang P.-S., Lee E.-F., Fan S.-K., Lu Y.-W. (2017). Genomic DNA extraction from whole blood using a digital microfluidic (DMF) platform with magnetic beads. Microsyst. Technol..

